# Supplementary material for: C9orf72 arginine-rich dipeptide repeat proteins disrupt karyopherin-mediated nuclear import
Source: eLife. 2020 Mar 2;9:e51685. doi: 10.7554/eLife.51685 (PMC7051184; doi:10.7554/eLife.51685)
Supplement: Transparent reporting form [file elife-51685-transrepform.pdf]

eLIFE
Westbrook Centre, Milton Road
Cambridge CB4 1YG
UK
P 01223 855340
W elifesciences.org
T @elife

eLife's transparent reporting form

We encourage authors to provide detailed information *within their submission* to facilitate the interpretation and replication of experiments. Authors can upload supporting documentation to indicate the use of appropriate reporting guidelines for health-related research (see EQUATOR Network), life science research (see the BioSharing Information Resource), or the ARRIVE guidelines for reporting work involving animal research. Where applicable, authors should refer to any relevant reporting standards documents in this form.

If you have any questions, please consult our Journal Policies and/or contact us: editorial@elifesciences.org.

Sample-size estimation

- You should state whether an appropriate sample size was computed when the study was being designed
- You should state the statistical method of sample size computation and any required assumptions
- If no explicit power analysis was used, you should describe how you decided what sample (replicate) size (number) to use

Please outline where this information can be found within the submission (e.g., sections or figure legends), or explain why this information doesn't apply to your submission:

Power calculations were not used to compute samples sizes for these studies. For active and passive nuclear transport and bead halo studies, biologically relevant positive and negative controls were embedded in all experiments to confirm the ability of the assay to detect meaningful differences. The nuclear transport experiments were done in 96-well format via automated high content analysis, which allowed us to sample hundreds to thousands of cells per well in an unbiased manner, far exceeding the number of cells needed to show biological differences with high confidence. For FRET, Western blot and mass spectrometry studies, a minimum of two technical and/or biological replicates was done as stated in the figure legends, to ensure that representative data are shown.

Replicates

- You should report how often each experiment was performed
- You should include a definition of biological versus technical replication
- The data obtained should be provided and sufficient information should be provided to indicate the number of independent biological and/or technical replicates
- If you encountered any outliers, you should describe how these were handled
- Criteria for exclusion/inclusion of data should be clearly stated
- High-throughput sequence data should be uploaded before submission, with a private link for reviewers provided (these are available from both GEO and ArrayExpress)

Please outline where this information can be found within the submission (e.g., sections or figure legends), or explain why this information doesn't apply to your submission:

eLife Sciences Publications, Ltd is a limited liability non-profit non-stock corporation incorporated in the State of Delaware, USA, with company number 5030732, and is registered in the UK with company number FC030576 and branch number BR015634 at the address Westbrook Centre, Milton Road Cambridge CB4 1YG, UK | March 2019

The number of biological and technical replicates for each experiment are listed in the figure legends and shown in detail in the accompanying source data. For experiments in permeabilized neurons and Hela, biological replicates are single 96 well plates that were individually permeabilized for transport reactions (no more than two plates were used from the same neuronal culture prep or HeLa passage). Technical replicates refer to duplicate wells within the same 96 well plate. In all cases, the number of biological replicates was used for statistical analysis. Bead halo studies involved a single replicate, with embedded positive and negative controls as shown. FRET assays are representative of multiple biological replicates (freshly combined from the same parent proteins). Western blot and mass spectrometry data are representative of two technical replicates (two analyses of the same parent sample).

Statistical reporting

- Statistical analysis methods should be described and justified
- Raw data should be presented in figures whenever informative to do so (typically when N per group is less than 10)
- For each experiment, you should identify the statistical tests used, exact values of N, definitions of center, methods of multiple test correction, and dispersion and precision measures (e.g., mean, median, SD, SEM, confidence intervals; and, for the major substantive results, a measure of effect size (e.g., Pearson's r, Cohen's d)
- Report exact p-values wherever possible alongside the summary statistics and 95% confidence intervals. These should be reported for all key questions and not only when the p-value is less than 0.05.

Please outline where this information can be found within the submission (e.g., sections or figure legends), or explain why this information doesn't apply to your submission:

Method of statistical analysis, tests used, and type of data depicted can be found in the figure legends. For the sake of space, ranges of sample size and replicates are given in the figure legends, and individually detailed in a supplemental table along with the exact p-values.

(For large datasets, or papers with a very large number of statistical tests, you may upload a single table file with tests, Ns, etc., with reference to sections in the manuscript.)

Group allocation

- Indicate how samples were allocated into experimental groups (in the case of clinical studies, please specify allocation to treatment method); if randomization was used, please also state if restricted randomization was applied
- Indicate if masking was used during group allocation, data collection and/or data analysis

Please outline where this information can be found within the submission (e.g., sections or figure legends), or explain why this information doesn't apply to your submission:

All nuclear transport analysis was done by automated high content imaging, including selection of regions of interest and nuclear/cytoplasmic ratio calculations. Bead halo image analysis was done by an investigator blinded to treatment group and experimental intent. These details are described in the materials and methods section.

Additional data files ("source data")

- We encourage you to upload relevant additional data files, such as numerical data that are represented as a graph in a figure, or as a summary table
- Where provided, these should be in the most useful format, and they can be uploaded as "Source data" files linked to a main figure or table
- Include model definition files including the full list of parameters used
- Include code used for data analysis (e.g., R, MatLab)
- Avoid stating that data files are "available upon request"

Please indicate the figures or tables for which source data files have been provided:

Raw data for all figures and supplemental figures in the manuscript have been provided in the source files.
